# Transport processes of radiopharmaceuticals and -modulators

**DOI:** 10.1186/1748-717X-6-59

**Published:** 2011-06-06

**Authors:** Thomas Efferth, Peter Langguth

**Affiliations:** 1 Department of Pharmaceutical Biology, Institute of Pharmacy and Biochemistry, Johannes Gutenberg-University, Mainz, Germany; 2 Department of Pharmaceutical Technology and Biopharmacy, Institute of Pharmacy and Biochemistry, Johannes Gutenberg-University, Mainz, Germany

**Keywords:** ABC transporter, Multidrug resistance, Radioresistance, Radioprotection, Radiochemotherapy

## Abstract

Radiotherapy and radiology have been indispensable components in cancer care for many years. The detection limit of small tumor foci as well as the development of radio-resistance and severe side effects towards normal tissues led to the development of strategies to improve radio-diagnostic and -therapeutic approaches by pharmaceuticals. The term "radiopharmaceutical" has been used for drugs labeled with radioactive tracers for therapy or diagnosis. In addition, drugs have been described to sensitize tumor cells to radiotherapy (radiosensitizers) or to protect normal tissues from detrimental effects of radiation (radioprotectors). The present review summarizes recent concepts on the transport of radiopharmaceuticals, radiosensitizers, and radioprotectors in cells and tissues, e.g. by ATP-binding cassette transporters such as P-glycoprotein. Strengths and weaknesses of current strategies to improve transport-based processes are discussed.

## Introduction

Together with surgery and chemotherapy, radiotherapy represents one of the main pillars of cancer therapy. The field of radiology for image-based cancer diagnostics experiences rapid progress in the past years making both radiotherapy and -diagnostic to indispensible components in cancer care.

A number of strategies have been developed to increase efficacy of radiotherapeutic and -diagnostic approaches by pharmaceuticals. This represents exciting interdisciplinary opportunities for research in medicine and physics on the one hand and pharmacy and pharmacology on the other hand.

Traditionally, the term "radiopharmaceutical" has been used for drugs labeled with radioactive tracers for therapeutic or diagnostic purposes. Due to the enormous progress in the past decade, the interface between drug treatment and radiotherapy became much broader. Many drugs have been described to sensitize tumor cells to radiotherapy or to protect normal tissues from radiation-induced injuries. In a broader sense, those drugs are also "radiopharmaceuticals". Even drugs which increase the efficacy of other forms of radiation-based therapy have to be named in this context, *e.g. *8-methoxypsoralen in UVA-therapy or enhancers of photodynamic drugs. In order to avoid confusion with the term "radiopharmaceutical" in the narrow sense, we propose the term radiomodulator for drugs sensitizing tumor cells or protecting normal tissues to all forms of radiation therapy.

The multiple and partly heterogeneous aspects of radiopharmaceuticals and -modulators can be separated into three major fields:

(1) **Radiopharmaceuticals **are used in nuclear medicine as tracers for diagnostics and therapy of many diseases. Technetium 99m (Tc-99m) serves as gamma-rays-emitting tracer nuclide for many radiopharmaceuticals. More than 30 different Tc-99m-based radiopharmaceuticals are known, which are used for imaging and functional studies in diverse organs, *e.g. *brain, lung, kidneys, liver, skeleton *etc*. [1]. They also serve for diagnostic visualization of tumors. Moreover, numerous radiopharmaceuticals have been developed with other radioisotopes than Tc-99m. Their localization in the body is also determined by gamma-ray measurement. Radioisotopes suitable for this purpose are Fluor-18, Gallium-67, Gallium-68, Jod-124 and many more. Another interesting treatment option is boron neutron capture therapy (BNCT) which is based on the neutron capture reaction of the stable isotope ^10^B by irradiating this isotope with thermal neutrons (E_n_<0.1 KeV), the ionized particles ^4^He and ^7^Li are generated from the ^10^B(n,α)^7^Li reaction [2]. Preloading of cells with specific markers can be used for the treatment of specific cancer types [3].

(2) **Radiosensitizers: **About one half of all patients with a solid tumor are treated by radiotherapy. The effectiveness of this treatment option is, however, frequently hampered by the development of radio-resistance [4]. Therefore, the combination of radiotherapy with drugs to sensitize tumors towards radiotherapy is an attractive strategy [5,6]. At the same time, the radiation effects on normal tissues should not be increased by radio-sensitizing agents.

Ionizing radiation causes DNA damage by the generation of reactive oxygen species (ROS), especially DNA double strand breaks. Diverse established anticancer agents have been described to sensitize tumors towards radiotherapy by interaction with DNA biosynthesis (5-fluorouracile, gemcitabine, hydroxyurea) [7] or inhibition of DNA-replication and repair by adduct formation (temozolomide, cisplatin) [8,9]. DNA topoisomerase inhibitors (topotecan, irinotecan) have also been shown to exert radio-sensitizing effects [10]. Mitotic spindle poisons (paclitaxel, docetaxel) arrest tumor cells in the G_2_M phase of the cell cycle[11].

Frequently, hypoxic areas are found in tumors. As oxygen is necessary for the formation of radical molecules, which are important in radiotherapy, hypoxic tumors are radio-resistant. Various strategies have been advised to overcome this problem, *e.g. *re-oxygenation of hypoxic tumors (nitroimidazole compounds, efaproxiral), the activation of intracellular reductases by bioreductive cell poisons (tirapazamine) or the inhibition of the hypoxia-inducing factor (HIF-1) [12,13]. HIF-1 inhibitors are more in the focus of interest, as nitroimidazoles reveal a narrow therapeutic window.

In the past years, rational radio-sensitizing concepts have been investigated, which are based on the inhibition of specific target proteins [14]. Examples are inhibitors of specific repair enzymes for radiation-induced damage (ATM kinase), DNA-PK, Chk1 and Parp-1 (*e.g. *caffeine, [15,16] as well as inhibitors of epidermal growth factor receptor (EGFR) pathway (cetuximab, erlotinib) and inhibitors of downstream signaling routes (wortmannin). Radio-sensitizing effects have also been observed by inhibitors of the NF-κB transcription factor and substances, which switch off radiation-induced apoptosis (p53 modulators, Bcl-2 inhibitors). Trichostatin A inhibits histone deacetylases and geldanamycin blocks the heat shock protein, HSP90. The suppression of blood vessel formation in tumors (neoangiogenesis) gained much attention in the past years. Interestingly, various angiogenesis inhibitors, *e.g. *blockers of the vascular endothelial growth factor (VEGF) also exert radio-sensitizing effects.

Special forms of radiosensitizers are photosensitizers, *i.e. *chemical compounds excited by visible or near-infrared light. If accumulated in tumors and illuminated by light, photo-sensitizers generate singlet oxygen destroying tumor cells [17-19]. Broadband ultraviolet B (BB-UVB), and psoralen plus and ultraviolet A (PUVA), and more recently narrowband UVB (NB-UVB) are skin-directed phototherapies used to treat cutaneous T-cell lymphoma. Extracorporeal photopheresis (ECP) is effective in more advanced stage disease [20-22].

In photodynamic therapy (PDT), photosensitizers such as photofrin are excited by light of a specific wavelength. Interestingly, some types of photosensitizers are substrates of Breast Cancer Resistance Protein (BCRP, ABCG2) leading to resistance of tumors to PDT [23]. For example, A431 lung cancer cells transfected with BCRP were more resistant to photofrin-PDT than A431 control cells in vitro and fumitremorgin C, a specific BCRP inhibitor, reversed this resistance [24]. A clinical study with 81 lung cancer patients showed that the efficacy of photofrin-PCT in cancer lesions was significantly affected by the expression of BCRP [24].

Genetic polymorphisms and transcriptional activation in the ABCG2 (BRCP) transporter influenced cellular accumulation of porphyrin derivatives in cancer cells leading to individual differences of patients in their response to photodynamic therapy [23].

(3) **Radioprotectors: **Protection from radiation-induced damage is important to

• avoid radiation damage in healthy tissue and organs during radiotherapy of tumors

• save personnel of airlines from excessive exposure to radiation in the air.

Various natural products and synthetic compounds have been described as radioprotectors [25]. They can be separated in four categories:

(a) Scavenger of ROS and other radical molecules. Selen and selenoproteins exert anti-oxidant, radio-protective and anti-carcinogenic effects. A possible effector of this radio-protective effect is glutathione peroxidase. This opens the possibility that nutritional supplementation with L-selenomethionine might represent a suitable radioprotector for airline personnel [26].

(b) Further radio-protective nutritional supplements are N-acetyl-L-cysteine (NAC), tocopherol succinate (a vitamin E analogue) and eugenol. Resveratrol and other polyphenols activate Sirt1 expression. Sirtuins are NAD^+^-dependent deacetylases, which interact with the NBS1 DNA repair protein, thereby regulating DNA damage repair. Furthermore, resveratrol suppresses inflammatory processes by inhibition of prostaglandin production, COX2 expression and NFκB activity. Resveratrol also induces G1 and G1/S cell cycle arrest and apoptosis [26,27].

(c) Manganese-dependent superoxide dismutase (MnSOD) is an anti-oxidant enzyme. Small molecule MnSOD inhibitors and gene-therapeutic strategies based on MnSOD have been described to lower cellular ROS levels for radio-protection [27].

(d) Amifostine and its active metabolite, WR-1065, are non-protein-thiols, scavenging ROS and other radical molecules. Thereby, they support DNA damage repair and influence intracellular hypoxia by auto-oxidation processes [27].

(e) Improvement of DNA damage repair and modulation of signal transduction after DNA damage. Amifostine also inhibits DNA topoisomerase II leading to the arrest of damaged cells in the G_2_M phase of the cell cycle. Thereby, the homologous recombination DNA repair pathway in the G_2_M phase is more effective [27].

(f) Inhibition of apoptosis in radiation-damaged cells. Flagellin is a natural product of bacteria. The flagellin derivative, CBLB502, activates NFκB via the toll-like receptor-5 (TLR-5) and inhibits the onset of apoptosis. The synthetic small molecule, PD 0332991 inhibits CDK-4 and -6 and protects from radiation damage by induction of Ras-mediated cellular quiescence and inhibition of apoptosis [28,29].

### Transport through bio-membranes

The first barrier for drugs represents the entry from the body surface to the body inside (absorption). In this context, the inside of the gastrointestinal tract is understood as body surface. The cellular structures separating the outside of the body from the inside are lipid bilayer-consisting cell membranes. Hence, the passage through bio-membranes is a precondition for drug activity.

This can happen by diverse mechanisms [30]:

(1) Lipophilic substances enter cell membranes by passive diffusion or passive transport (carrier) without energy, *i.e*. ATP consumption.

(2) Hydrophilic compounds enter cell membranes by passive transport (e.g. ion channels) or active transport (ATP-consuming transport).

(3) For vesicular transport, extracellular compounds are included into vesicles, which constrict into the intracellular space (phagocytosis, pinocytosis).

(4) For receptor-mediated endocytosis, compounds bind to receptors on the cell surface. Receptor-ligand-complexes are accumulated in coated pits of the cell membrane and are internalized by endocytosis.

There are three super-families of ATP-consuming transporters with eminent relevance for drug transport:

(1) ATP-binding cassette (ABC) transporter. This family consists of 49 members in the human genome [31-33]. The multidrug resistance-mediating transporters P-glycoprotein (*ABCB1, MDR1*), multidrug resistance-related proteins (ABCC, MRP), and the breast cancer resistance protein (ABCG2, BCRP) belong to this family [34,35]. They confer resistance towards anticancer drugs in tumors. In healthy tissues and organs they have a protective function towards xenobiotic compounds, *e.g. *in the blood-brain-barrier [36].

(2) The solute carrier (SLC) superfamily contains members with very diverse functions. The subfamilies, SLC6A, 10A, 15A, 16A, 17A, 22A and 29 A are associated with the transport of xenobiotics. These subfamilies consist of organic anion transporters, organic cation transporters, nucleoside transporters, amino acid and peptide transporters [37].

(3) The sodium-independent large organic anion transporters (SLCO) are a third superfamily involved in drug transport. Previously, they were assigned to the SLC superfamily as SLC21A subfamily, but now they consist of an own gene family [38,39].

In addition to drug uptake, the distribution in tissues and organs are essential for drug activity. Drugs are frequently bound to transfer proteins. As an example, albumin binds many different free drug molecules in the blood. Moreover, there are more specific transfer molecules, which only bind certain drug classes, *e.g*. α-tocopherol-binding proteins or transferrin, which binds iron and enables its uptake into cells [40,41].

**Figure 1:**
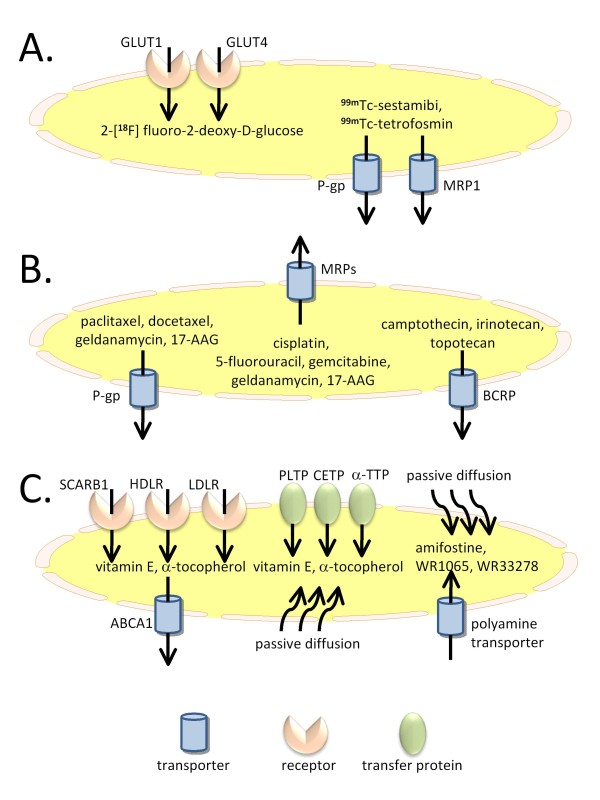
**Synopsis of cellular transport processes**. (A) Transport of radiopharmaceuticals; (B) transport of radiosensitizers; (C) transport of radiomodulators. Abbreviations: 17-AAG, 17-allylamino-demethoxygeldanamycin; α-TTP, alpha-tocopherol transfer protein; ABCA1, ATP-binding cassette transporter A1; BCRP, breast cancer resistance protein; CETP, colesterylester transfer protein; GLUT1/4, glucose transporter 1/4; HDLR, high density lipid receptor; LDLR, low density lipid receptor; MRP, multidrug resistance-related protein; P-gp, P-glycoprotein; PLTR, phospholipid transfer protein;, SCARB1, scavenger receptor-class BI-

Unambiguously, drug transport is of eminent importance for drug activity. This is true for radiopharmaceuticals as well as radiomodulators. There was a thriving development in this field in the past years, which is a fertile ground for exciting novel research concepts in the years to come. A synopsis of cellular transport processes described in this review is depicted in Figure 1.

### Transport of radiopharmaceuticals

**Figure 2:**
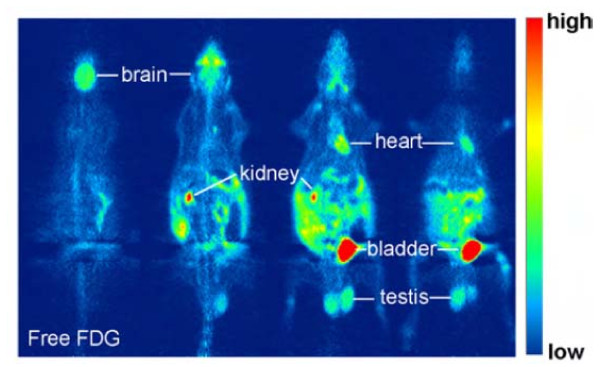
**Whole body images 2 h after i.p. injection (horizontal slices, thickness 7 mm)**. Free [^18^F]FDG is taken up by high-glucose using cells such as brain, heart and testis and excreted via kidney and bladder. Liposomal encapsulated [^18^F]FDG accumulates in the abdomen of the rat. Only released [^18^F]FDG can be taken up into organs (Picture taken from [41]).

A well-known radiotracer for positron emission tomography (PET) is 2-[^18^F] fluoro-2-deoxy-D-glucose (FDG) (Figure 2). Tumor imaging by FDG-PET is based on a fundamental observation of Otto Warburg in the first half of the 20^th ^century. He found that cancer cells take up more glucose than normal cells. Despite reduced oxygen consumption, they have higher glycolysis rates [42]. FDG is taken up by tumor cells via the glucose transporters, GLUT1 and GLUT4. Intracellularly, FDG is phosphorylated to FDG-6-phosphate without further metabolization. Thereby, it is accumulated more in tumors than in normal surrounding tissues. Tumor cells express more GLUT1 than normal cells.

Though FDG-PET is already established in clinical routine diagnostics [43], there are several problems. The GLUT1 expression largely varies from tumor type to tumor type affecting FDG uptake. Proliferation rate, hypoxia, inflammatory infiltrates and even blood sugar level all influence FDG uptake. Hence, there is an urgent need for novel radiotracers with more specific target properties [44].

The sodium-iodide symporter (NIC) uses the sodium gradient built up by sodium-potassium ATPase in the cell membrane for the co-transport of iodide and sodium into the cell. This co-transport can be especially observed in the pituitary, lactating breast epithelia as well as sweat glands and in stomach mucosa [45]. Therefore, these tissues accumulate the radioactive isotopes, ^123^I and ^131^I as well as ^99m^Tc-pertechnetates. This can be used in nuclear medicine for diagnosis and therapy of pituitary diseases [46].

P-glycoprotein (*ABCB1, MDR1*) is well-known in terms of its function for multidrug resistance of tumors [31,33]. Two major therapy concepts have been developed with P-glycoprotein as target molecule:

• Pharmacological inhibition of P-glycoprotein and, thereby, the re-sensitization of multidrug-resistance

• *MDR1*- based gene therapy of healthy bone marrow cells to confer resistance towards high-dose chemotherapy. High concentrations of anticancer drugs would kill tumor cells but spare healthy bone marrow because of the gene therapeutic *MDR1 *expression.

For both concepts, non-invasive molecular imaging techniques are desired to predict and monitor treatment success. For this purpose, various γ-ray-emitting substances have been developed, which are transport substrates of P-glycoprotein. The best-known radiopharmaceuticals in this context are ^99m^Tc-sestamibi [47,48] and ^99m^Tc-Tetrofosmin [49]. P-glycoprotein-expressing multidrug-resistant tumor cells extrude both compounds out of the cell whereas P-glycoprotein-negative, drug-sensitive tumor cells accumulate the substances. This uptake can be visualized as hot spots by scintigraphy. The realization of this strategy has been demonstrated in several proof-of-principle clinical trials. The conclusion of these studies was that both substances are able to predict multidrug-resistance of tumors caused by P-glycoprotein [50-53]. ^99m^Tc-sestamibi revealed P-glycoprotein specificity in cancer patients. An MRP1-related transport of this compound could not be detected, although this was observed in preclinical models *in vitro *[49,54]. ^99m^Tc-Tetrofosmin was also found to be transported by MRP1 *in vitro *[54,55]. In a clinical trial, ^99m^Tc-MIBI accumulation in patients correlated with the absence of both P-glycoprotein and MRP1 expression, indicating that ^99m^Tc-MIBI is also transported by MRP1 [56]. Similar results have been found for ^99m^Tc-Tetrofosmin in lymphoma patients [57].

### Transport of radiosensitizers

Many established anticancer agents used to sensitize radiation effects in combined radiochemotherapy are substrates of drug transporters. Mitotic spindle poisons such as paclitaxel and docetaxel are well-known substrates of P-glycoprotein. Camptothecin derivatives (topotecan, irinotecan) are transported by BCRP (ABCG2). Multidrug resistance-related proteins of the ABCC subfamily of ABC transporters confer resistance towards cisplatin, 5-fluorouracil and gemcitabine. Moreover, copper transporters (*e.g*. CTR1) and volume-sensitive chloride channels also contribute to cisplatin resistance. Changes of membrane fluidity are a further factor of drug resistance. In as much as transport processes influence drug resistance, they also affect radio-sensitizing effects in combined radiochemotherapy, since necessary drug levels are not reached in tumors.

Ionizing radiation has been shown to enhance cellular resistance to anticancer drugs, including methotrexate, 6-thioguanine, cisplatin and others [58,59]. Hill et al. were the first to report that in vitro exposure of mammalian cells to fractionated irradiation results in the expression of a multidrug resistance phenotype with cross-resistance to *Vinca *alkaloids, epipodophyllotoxins and colchicine, but not to anthracyclines [60]. These cell lines revealed an overexpression of P-glycoprotein, but not of MDR1 mRNA. Studies to elucidate the reason for increased protein but unchanged mRNA levels upon X-irradiation revealed a slower turnover of P-glycoprotein in X-irradiated cells(half life: 40 h) relative to classical drug-selected sublines (half life: 17 h), indicating that P-glycoprotein gp overexpression may be differently regulated in these sublines at the translational level [61]. These data clearly show that the development of drug resistance following X-irradiation arise by a mechanism distinct from that operating after drug selection. Fractionated irradiation of a human epidermoid lung carcinoma xenograft grown in nude mice also results in overexpression of P-glycoprotein without concomitant MDR1 mRNA overexpression [62]. This in vivo approach mimics the clinical situation and points to a possible role of irradiation for P-glycoprotein overexpression and induction of drug resistance in radiochemotherapy. Subsequently, similar results have been found for MRP1. Fractionated X-irradiation increased resistance of tumor cell lines to anticancer drugs and induced expression of MRP1 [63-65].

Nitroimidazoles have been coupled to sugar molecules, since hypoxic tumors reveal higher glycolysis and higher membrane transporter-mediated glucose uptake [66]. Hybrid molecules of sugars and nitroimidazoles (*e.g. *TX-2224) showed increased cellular uptake and at the same time radio-sensitizing activity [67]. Similarly, ifosfamide has been coupled to sugars (glufosfamide) to sensitize hypoxic tumors for radiochemotherapy [68]. Increased glucose uptake in hypoxic and radio-resistant tumors is also the rationale for using 2-deoxy-D-glucose (2-DG). This compound inhibits the first enzyme of glycolysis (hexokinase), increases metabolic oxidative stress in tumor cells and sensitizes tumor cells towards radiation [69,70]. Many natural products have been described to exert radio-sensitizing effects and affect function and expression of transport proteins at the same time. For instance, the radiosensitizing trichostatin A down-regulates P-glycoprotein (*MDR1) *expression [71].

On the other hand, over-expression of *MDR1 *or *MRP1 *confers resistance towards geldanamycin and its derivative, 17-allylamino-demethoxygeldanamycin (17-AAG) [72,73]. Both compounds reveal radio-sensitizing effects by inhibition of the heat shock protein, HSP90.

Wortmannin inhibits the P-glycoprotein function [74], and a wortmannin-dependent PI3K/Akt inhibition correlates with reduced MRP1 expression [75]. Interestingly, wortmannin also inhibits the insulin-induced activation of the GLUT4 glucose transporter [76]. Glucose transporters are of prognostic value as reported for ovarian carcinoma [77]. Poorly differentiated tumors showed a trend to over-express the GLUT1 protein compared with the more differentiated counterparts. Patients who experienced a complete clinical response to chemotherapy were more frequently GLUT1 positive than GLUT1 negative. In multivariate analysis of advanced stage disease, residual tumor and high GLUT1 expression levels were the only independent variables that maintained a significant association with response to chemotherapy. In Stage III-IV patients showing a complete clinical response, GLUT1 over-expression was associated with a shorter disease-free survival.

Resveratrol and many other polyphenolic compounds inhibit P-glycoprotein and other ABC transporters in terms of function and expression [78,79]. The fact that resveratrol and other flavonoids act as inhibitors of P-glycoprotein raised much interest, because clinical trials with synthetic compounds to modulate the function of P-glycoprotein were not very promising yet [80,81]. Most of these resistance-modifying agents are too toxic at the required doses. Therefore, the search for P-glycoprotein inhibitors from the field of natural products may be more promising, since many natural products and phytotherapeutics are appreciated for their low side effects and good tolerability. As P-glycoprotein detoxifies xenobiotic compounds in normal tissues taken up with food, it can be expected that many herbal compounds are substrates of this efflux transporter. Indeed, there is a large body of evidence that natural compounds are transported by P-glycoprotein. From an evolutionary point of view, substrates and inhibitors of P-glycoprotein have been frequently co-developed in the same plant species. Plants developed secondary metabolites during evolution of life to defend against predators such as herbivores. If herbivores detoxify harmful natural products by P-glycoprotein, plants need inhibitors of P-glycoprotein for efficient self-defense. Hence it can be speculated that many P-glycoprotein inhibitors should be present in plants [82]. During the past years, P-glycoprotein-inhibiting activities have frequently been observed. The large number of data can be separated in two major categories: natural products either functionally inhibit P-glycoprotein by interference with efflux activity of the drug pump or they down-regulate P-glycoprotein/*MDR1 *expression, thereby re-sensitizing multidrug-resistant cells [83]. Some of these compounds inhibit not only P-glycoprotein, but also MRP1 [84-86] or BCRP [86-88]. It has not yet unequivocally been clarified, whether large amounts of polyphenols taken up with fruits and vegetables may influence absorption, distribution, and secretion of drugs in general or radiosensitizers.

### Transport of radioprotectors

The use of radioprotective agents in cancer therapy raises the question, how normal tissues, but not tumors can selectively be protected. Specific transport process may be helpful for the development of such strategies.

Amifostine (WR-2721) and its derivatives are phosphoaminothionates. As pro-drugs, they are dephosphorylated by alkaline phosphatase. The metabolite, WR 1065, passively diffuses through the cell membrane and is oxidized to a disulfide, WR-33278. As this compound reveals chemical similarity to polyamine spermine, it is then transported by the ornithine decarboxylase (ODC)-antizyme (*OAZ*)-dependent polyamine transporter leading to cellular accumulation of amifostine derivatives [90,91]. A selective radioprotection of normal but not tumor cells may be achieved by transfection of *OAZ *cDNA. Thereby, the polyamine transporter and, hence, WR-33278 uptake is inhibited. Since tumor cells reveal higher ODC activities and polyamine contents as normal tissues, a combination therapy of amifostine and *OAZ *gene transfer may result in an increased eradication of tumor cells with protection of normal tissues at the same time [92].

The topical application of radioprotectors in the form of creams and ointments for airline employees, depends on dermal absorption. The passage through the Stratum corneum to the epidermis and dermis is not only influenced by the radio-protecting agent itself, but also by the formulation, as shown for amifostine (WR-2721) [93].

**Figure 3:**
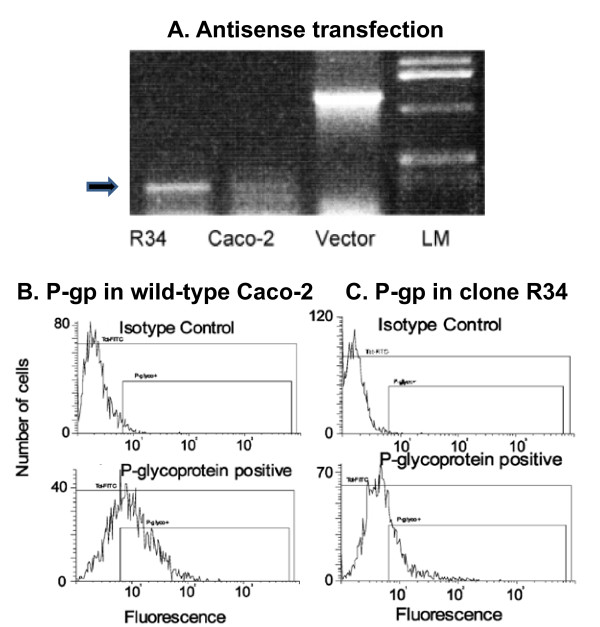
***MDR1 *antisense-mediated down-regulation of P-glycoprotein (P-gp)**. (A) RT-PCR reactions on mRNA from wild-type Caco-2 cells and Caco-2 subclone R34, generated by transfection with *MDR1 *antisense gene cloned into the pEUK-c1 vector. A specific band (arrow) of antisense *MDR1 *mRNA was found in clone R34. Parental Caco-2 cells did not show this amplification product. The positive control PCR on the pEUK-c1-RDM vector gives a product 965 bases longer, as the vector contains an intron. (LM) ladder marker, representing different molecular lengths). FACS analysis of (B) wild-type Caco-2 cells and (C) *MDR1*-antisense transfectants R34 for the expression of P-glycoprotein. The cells were incubated with an non-specific mouse-antibody and FITC-labeled anti-mouse-antibody (isotype control), and specifically labeled with the monoclonal antibody MRK16 and FITC-labeled anti-mouse-antibody (P-gp expression). Isotype control analysis did not reveal significant numbers of cells that would be considered P-gp positive. Compared to non-transfected cells (79.4%), the P-glycoprotein expression in clone R34 was significantly reduced (41.8%) (Pictures taken from [73]).

Remarkably, radiosensitivity also depends on the expression of drug transporters independently of a simultaneous application of radio-protective or radio-sensitizing agents. The retroviral transfer of the *MDR1 *gene induced differential expression of genes, including up-regulation of detoxifying and down-regulation of pro-apoptotic genes [94]. Hence, it can be speculated that *MDR1*-based gene therapy as well as induction of *MDR1 *gene expression by chemical agents (*e.g*. small molecules) may favor the protection of normal tissues from radiation-induced damage [95] (Figure 3).

The efficacy of radiochemotherapy is also determined by transporter-independent processes. In response to gamma-ray, whole body irradiation, changes in the intestinal membrane fluidity and lipid peroxidation have been observed [96]. Membrane fluidity is an important factor for the cellular uptake and accumulation of drugs.

Vitamin E and its main isomer, α-tocopherol are very hydrophobic and cannot be freely distributed in the blood stream. Rather, they associate with lipoproteins sharing many features with lipoprotein metabolism and cholesterol transport [97,98]. In addition to passive diffusion in the colon, active uptake by the transmembrane glycoprotein, scavenger receptor-class BI (SCARB1, SRBI), plays a role. The microsomal triglyceride transfer protein is required for enterocytic secretion of vitamin E into chylomicrons. Apparently, different transport systems are necessary for tissue distribution. In addition to SR-BI, tocopherol-associated proteins (TAP), phospholipid transfer protein (PLTP) and various other transport and transfer proteins of the lipid and cholesterol metabolism have been described, *e.g*. the ABCA1 transporter, the cholesterylester transfer protein (CETP), LDL-and HDL- receptors and others. SR-BI also plays a role for the selective α-tocopherol uptake across the blood brain barrier and the blood-retina-barrier. The hepatic uptake occurs *via *the α-tocopherol transfer protein (α-TTP).

The excretion takes place via bile and urine. ABC transporters in the canalicular membranes of hepatocytes contribute to biliary excretion. α-tocopherol is excreted as carboxyethyl-hydroxychromane.

## Conclusions and Perspectives

Taking the research progress in this field into consideration, a number of issues appear that have not adequately been addressed as yet:

(1) The relationships between drug and radio-resistance are incompletely understood. The elucidation of underlying molecular mechanisms is necessary to take advantage of synergistic effects for tumor therapy and of antagonistic effects to protect healthy tissues. Transport process plays an eminent role in this context. Solid data are available for P-glycoprotein providing evidence for the relevance of transport processes of radiopharmaceuticals and -modulators. The vast majority of other transport proteins have been scarcely investigated and their role for radiotherapy and radiochemotherapy is not understood yet.

(2) Many single results provide strong evidence for the importance of transport processes for radiopharmaceuticals and-modulators. However, a systematic synopsis integrating and validating existing single results is still missing. This may, however, be relevant for the realization of novel diagnostic and therapeutic strategies.

(3) The translation of results of basic research to the clinical everyday routine has to be considerably improved and accelerated from our point of view. The radiotracer and P-glycoprotein substrate Tm^99^-sestamibi is a suitable example to illustrate the clinical relevance of transport processes of radiopharmaceuticals. There is an enormous potential for other applications in radiology and nuclear medicine based on drug transport. The inter-connection of theoretical and experimental expertise in this field represents a critical mass to foster the progress for novel diagnostic and therapeutic approaches.

## Competing interests

The authors declare that they have no competing interests.

## Authors' contributions

TE and PL equally wrote this review article. Both authors read and approved the final manuscript.
